# Effects of dopamine D_1_ receptor blockade on the ERG b- and d-waves during blockade of ionotropic GABA receptors

**DOI:** 10.1186/s40662-016-0064-4

**Published:** 2016-12-07

**Authors:** Elka Popova, Momchil Kostov, Petia Kupenova

**Affiliations:** Department of Physiology, Medical University of Sofia, Sofia, Bulgaria

**Keywords:** Dopamine, D_1_ receptors, GABA, Electroretinogram, b-wave, d-wave

## Abstract

**Background:**

Some data indicate that the dopaminergic and GABAergic systems interact in the vertebrate retina, but the type of interactions is not well understood.

**Methods:**

In this study we investigated the effect of dopamine D_1_ receptor blockade by 75 μM SCH 23390 on the electroretinographic ON (b-wave) and OFF (d-wave) responses in intact frog eyecup preparations and in eyecups where the ionotropic GABA receptors were blocked by 50 μM picrotoxin. Student’s *t*-test, One-way repeated measures ANOVA with Bonferroni post-hoc test and Two-way ANOVA were used for statistical evaluation of the data.

**Results:**

We found that SCH 23390 alone significantly enhanced the amplitude of the b- and d-waves without altering their latency. The effect developed rapidly and was fully expressed within 8-11 min after the blocker application. Picrotoxin alone also markedly enhanced the amplitude of the ERG ON and OFF responses and increased their latency significantly. The effect was fully expressed within 25-27 min after picrotoxin application and remained very stable in the next 20 min. The effects of SCH 23390 and picrotoxin are similar to that reported in our previous studies. When SCH 23390 was applied on the background of the fully developed picrotoxin effect, it diminished the amplitude of the b- and d-waves in comparison to the corresponding values obtained during application of picrotoxin alone.

**Conclusion:**

Our results demonstrate that the enhancing effect of D_1_ receptor blockade on the amplitude of the ERG b- and d-waves is not evident during the ionotropic GABA receptor blockade, indicating an interaction between these neurotransmitter systems in the frog retina. We propose that the inhibitory effect of endogenous dopamine mediated by D_1_ receptors on the ERG ON and OFF responses in the frog retina may be due to the dopamine-evoked GABA release.

**Electronic supplementary material:**

The online version of this article (doi:10.1186/s40662-016-0064-4) contains supplementary material, which is available to authorized users.

## Background

Dopamine is the most abundant catecholamine in the vertebrate retina, while gamma-aminobutyric acid GABA is the major retinal inhibitory neurotransmitter. Dopamine is released by a specific set of dopaminergic amacrine and/or interplexiform cells (reviews: [1, 2]). Its actions are mediated by two classes of metabotropic G protein-coupled receptors: 1) D_1_-class receptors (including D_1_ and D_5_), which generally stimulate the production of cAMP and protein kinase A (PKA) activity and 2) D_2_-class receptors (including D_2_, D_3_ and D_4_), which generally decrease the production of cAMP and PKA activity. GABA is released by a large population of GABAergic neurons identified as amacrine, interplexiform and horizontal cells (review: [3]). The actions of GABA are mediated by 2 types of receptors: 1) ionotropic GABA_A_ and GABA_ρ_ (GABA_C_) receptors, which are ligand gated chloride channels and 2) metabotropic G protein-coupled GABA_B_ receptors that regulate adenylyl cyclase, voltage-gated Ca^2+^ channels or inwardly rectifying K^+^ channels. One of the most intriguing questions in retinal physiology is how these two important neurotransmitter systems interact and what are the consequences of these interactions for visual information processing?

A few studies were undertaken in order to investigate the functional effects of dopamine on the retinal GABAergic system. Some authors reported that dopamine and D_1_ receptor agonist SKF 38393 increased the GABA-induced currents in rat retinal amacrine cells through phosphorylation of GABA_A_ receptors by PKA [4]. Others demonstrated that dopamine and D_1_ receptor agonist SKF 38393 selectively reduced the GABA_C_ receptor current in catfish cone horizontal cells. The effect was mimicked by an adenylyl cyclase activator forskolin and blocked by the dopamine antagonist haloperidol [5]. Dopamine and D_1_ receptor agonist SKF 38393 relieved the GABAergic inhibition, mediated mainly (but not entirely) by GABA_C_ receptors, of calcium entry in most bipolar cell terminals in the tiger salamander retina [6]. As a consequence of this effect, dopamine relieved the GABAergic inhibition on the activity of third order retinal neurons. Consistent with these results is a recent finding that dopamine exerts a direct noncompetitive inhibition of human GABA_ρ_, but not GABA_A_ receptors, expressed in *Xenopus laevis* oocytes [7]. The authors proposed that “the binding site for dopamine in GABAρ_1_ receptor is different from the GABA binding site, and is probably not located inside the channel pore”. Thus, it appears that dopamine may have opposite effects on the currents mediated by GABA_A_ and GABA_ρ_ receptors in single retinal neurons.

One of the easiest ways to investigate the global retinal function in vivo without perturbing any neuronal connections is through the use of an electroretinogram (ERG). The ERG consists of many components, but two components are most prominent in response to long lasting stimuli: b-wave (in response to stimulus onset) and d-wave (in response to stimulus offset). The b- and d-waves are usually used for assessment of the retinal ON and OFF channel activity. There is general consensus that the neuronal generator of the b-wave is primarily the depolarizing (ON) bipolar cells, while the d-wave generation depends mainly on the activity of hyperpolarizing (OFF) bipolar cells with minor contribution of the photoreceptor response at stimulus offset and activity of proximal retinal neurons (reviews: [8, 9]). Therefore, the significance of the dopamine-GABA system interactions for the global retinal function could be revealed by investigating the ERG changes during manipulation (pharmacological or genetic) of the two systems. It has been shown that both GABA_C_R^-/-^ and D_1_R^-/-^ knockout mice have smaller b-wave amplitude, reduced dark sensitivity and compressed operational range of the rod-driven b-wave, while the blockade of GABA_A_ receptors did not change these parameters of the response [10]. The GABA injections into D_1_R^−/−^ mice restored the light sensitivity and operational range of b-waves, showing that the lack of D_1_R-mediated signaling can be completely masked by exogenous GABA. On the other hand, blockade of GABA_C_ receptors had little additive effect on the b-wave phenotype in D_1_R^−/−^ mice. Hence, the authors proposed that dopamine acting on D_1_-class receptors induces GABA release from horizontal cells and/or amacrine cells and that GABA is responsible for dopamine effects on the ERG b-wave. Smith et al. [11] argued, however, that the activation of dopamine D_1_-class receptors suppressed rather than facilitated GABAergic modulation of mice rod b-wave. They demonstrated that D_1_ receptor antagonist SKF 83566 and GABA_C_ receptor antagonist TPMPA as well as D_1_ receptor agonist SKF 38303 had similar suppressive effects on the rod-mediated b-wave amplitude and sensitivity. Isolating GABA_C_ receptor input to ON-bipolar cells (with gabazine and strychnine) reversed the effect of D_1_ receptor blockade on the amplitude and sensitivity of the b-wave indicating that D_1_ receptors can suppress GABA_C_ input to ON-BCs. The authors suggested that the D_1_ receptor block disinhibited GABA_A_ input to GABAergic inhibitory cells that synapse onto ON BCs GABA_C_ receptors. The above cited studies provide no information concerning the effects of dopamine on the GABAergic input to OFF bipolar cells because they used brief light flashes, which did not allow separation of ERG ON and OFF responses.

There are no ERG data with respect to dopamine-GABA interactions in the non-mammalian retina. In this study we investigated the effect of dopamine D_1_ receptor blockade with SCH 23390 on the ERG b- and d-waves in intact frog eyecup preparations and in eyecups where the ionotropic GABA receptors were blocked by picrotoxin. We found that the enhancing effect of SCH 23390 on the amplitude of the both ERG waves is not evident during the GABAergic blockade.

## Methods

The experiments were carried out on dark adapted eyecup preparations of the frog (*Rana ridibunda*), continuously superfused with Ringer solution (NaCl 110 mM, KCl 2.6 mM, NaHCO_3_ 10 mM, CaCl_2_ 1.6 mM, MgCl_2_ 0.8 mM, Glucose 2 mM; HCl 0.5 mM to adjust pH to 7.8) at a rate of 1.8 – 1.9 ml/min, at 16-18 °C and supplied with moistened O_2._ The frogs were first anesthetized in water containing 500 mg/l Tricaine methanesulfonate (Sigma-Aldrich Chemie GmbH). They were then decapitated and pithed. The experimental procedure has been approved by protocol № 8/15.04.2015 from the Committee for ethics in scientific research of Medical University of Sofia, Bulgaria. The D_1_-class dopamine receptors were blocked by using SCH 23390 (Sigma-Aldrich Chemie GmbH) dissolved in Ringer solution to a concentration of 75 μM. This concentration was chosen among other concentrations tested (25 μM and 50 μM) because it had maximal effect on the amplitude of both ERG waves (see Additional file 1). The ionotropic GABA_A_ and GABA_ρ_ receptors were blocked by using 50 μM picrotoxin (Flika, Buchs, Switzerland) dissolved in the perfusion solution. The same concentration of picrotoxin has been used in many of our previous studies [12–14]

### Experimental procedure

The frogs were dark adapted for 24 h and then the eyecups were prepared under dim red light. The rhythmic light stimulation began after additional period of dark adaptation for 10 min. Diffuse white light stimuli (150 W tungsten halogen lamp) with 5 s duration were presented repeatedly at interstimulus interval of 25 s. The light intensity was 6 × 10^2^ quanta s^-1^ μm^-2^ falling at the plane of the retina. The stimulus intensity was chosen on the basis of our previous experiments showing that it generates responses in the steepest part of the V – log I function for both the b- and d-waves in dark adapted frog eyecups [12, 15, 16]. Using of long stimuli may light adapt the retina, but it is not expected to change the drug effects because it has been shown in many of our previous works that the effects of SCH 23390 and PT did not depend critically on the state of light adaptation [12, 14, 15].

The results are based on 33 experiments, divided into 4 experimental groups according to the substances applied: 1) *Control group* (*n* = 8). The eyecups were perfused with Ringer solution throughout the whole 55 min period. 2) *SCH 23390 group* (*n* = 8). The eyecups were perfused with Ringer solution for 10 min first and then with 75 μM SCH 23390 for 25 min. In one of the experiments the perfusion was switched again to Ringer solution for 15 min in order to follow the recovery from the SCH 23390 effects. 3) *Picrotoxin group* (*n* = 9). The eyecups were perfused with Ringer solution for 10 min first and then with 50 μM picrotoxin for 45 min. The recovery from picrotoxin effects was not investigated in the present study because it has been demonstrated in our previous work [13]. 4) *Picrotoxin + SCH 23390 group* (*n* = 8). The eyecups were consequently perfused with Ringer solution (for 10 min), 50 μM picrotoxin (for 27 min) and 50 μM picrotoxin + 75 μM SCH 23390 (for 18 min).

### ERG recording and data analysis

The electroretinograms were recorded by means of non-polarized Ag/AgCl electrodes at bandpass of 0.1 - 1000 Hz (DC/AC differential amplifier model 3000; A-M Systems) and digitized at 2000 Hz, 16 bit resolution (Data acquisition system Biopac MP 150). The amplitude of the b-wave was measured from the peak of the a-wave to the peak of the b-wave, while that of the d-wave was measured from the baseline to the peak of the wave. Then the amplitudes were normalized to the values obtained in the 10th minute from the beginning of the experiments because it was the last minute of the control period. The latency of the ERG waves was measured from the stimulus onset (for b-wave) or offset (for d-wave) to the beginning of the wave. The latency was measured in the following time points from the beginning of the experiment: 10th minute - last minute of the control period; 21st minute – time when the SCH 23390 effects reached their maximum; 37th minute – time when the picrotoxin effects reached their maximum and it was the last minute prior to switching the perfusion to picrotoxin + SCH 23390; 48th minute – time when the SCH 23390 effects were expected to reach their maximum during combined picrotoxin + SCH 23390 application.

Two-way ANOVA (OriginPro 8 software, OriginLab Corporation, Northhampton, MA) was used for statistical evaluation of the differences in the normalized amplitude values between different groups. One-way repeated measures ANOVA with Bonferroni post-hoc test was used to evaluate the period where the blocker effect on the ERG wave amplitude was stable in time (plateau period). Paired *t*-test was used for statistical evaluation of the latency changes in each group at the above mentioned time points (compared to the 10th minute). A *p* value of <0.05 was considered significant.

## Results

### Control group

**Fig. 1:**
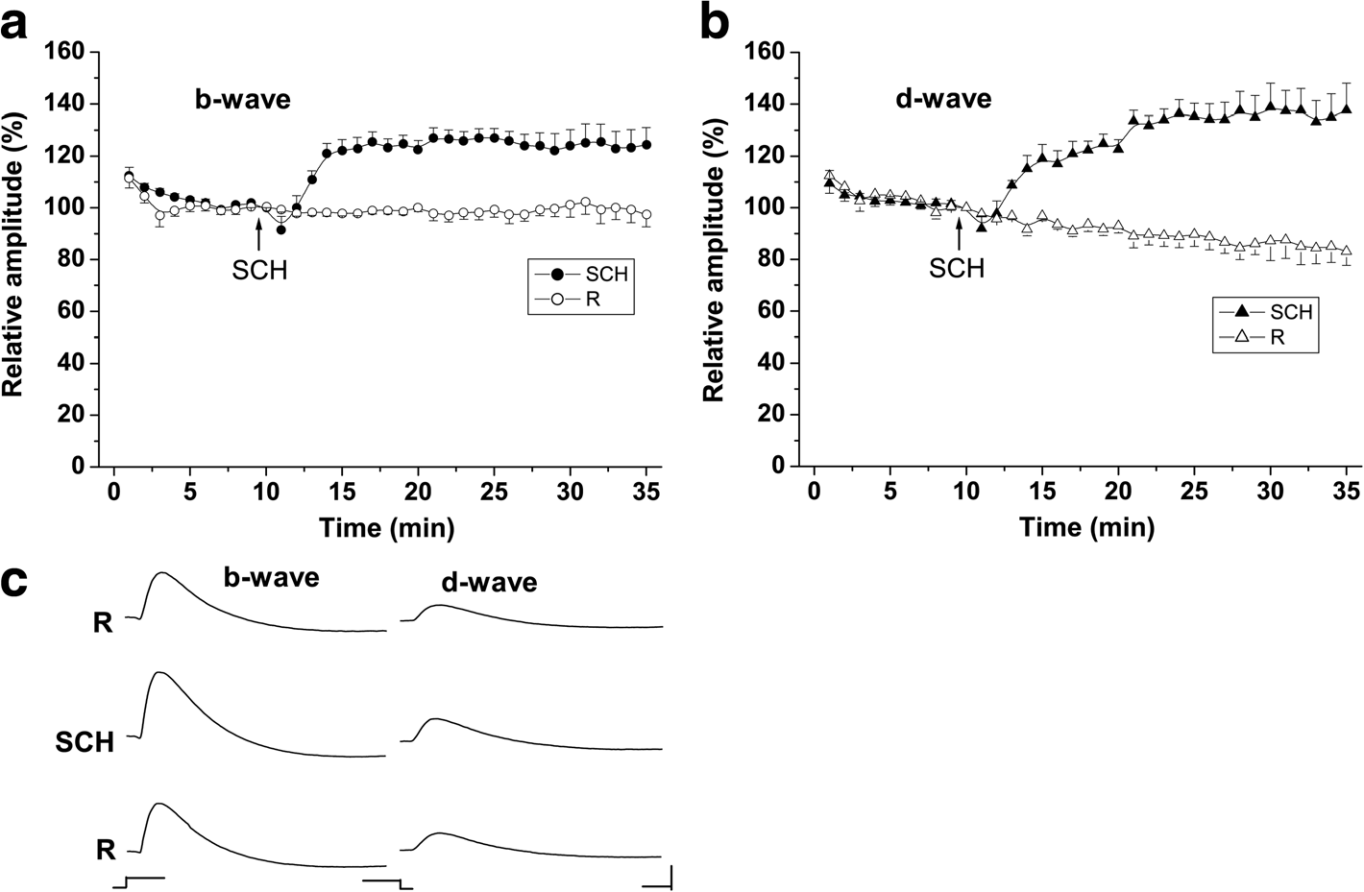
Effects of SCH 23390 on the ERG waves **a**, **b** Time course of the effects of SCH 23390 on the amplitudes of the ERG b- and d-waves. Results of both control experiments (R, open symbols) and test experiments (SCH, filled symbols) are represented. The amplitudes of the ERG waves were normalized to the values obtained in the 10th minute from the beginning of the experiments. The time when the perfusion was switched to 75 μM SCH 23390 (SCH) are indicated by arrows. Mean values ± SEM are shown. **c** Original ERG records (b- and d-wave) obtained during the perfusion with Ringer solution (R) in the control period (10th minute from the beginning of the experiment - upper row), 75 μM SCH 23390 (21st minute from the beginning of the experiment - middle row) and Ringer solution (R) in the recovery period (50th minute from the beginning of the experiment - bottom row). The stimulus onset and offset are indicated below the traces. Calibration: time – 0.2 s; amplitude – 50 μV

**Fig. 2:**
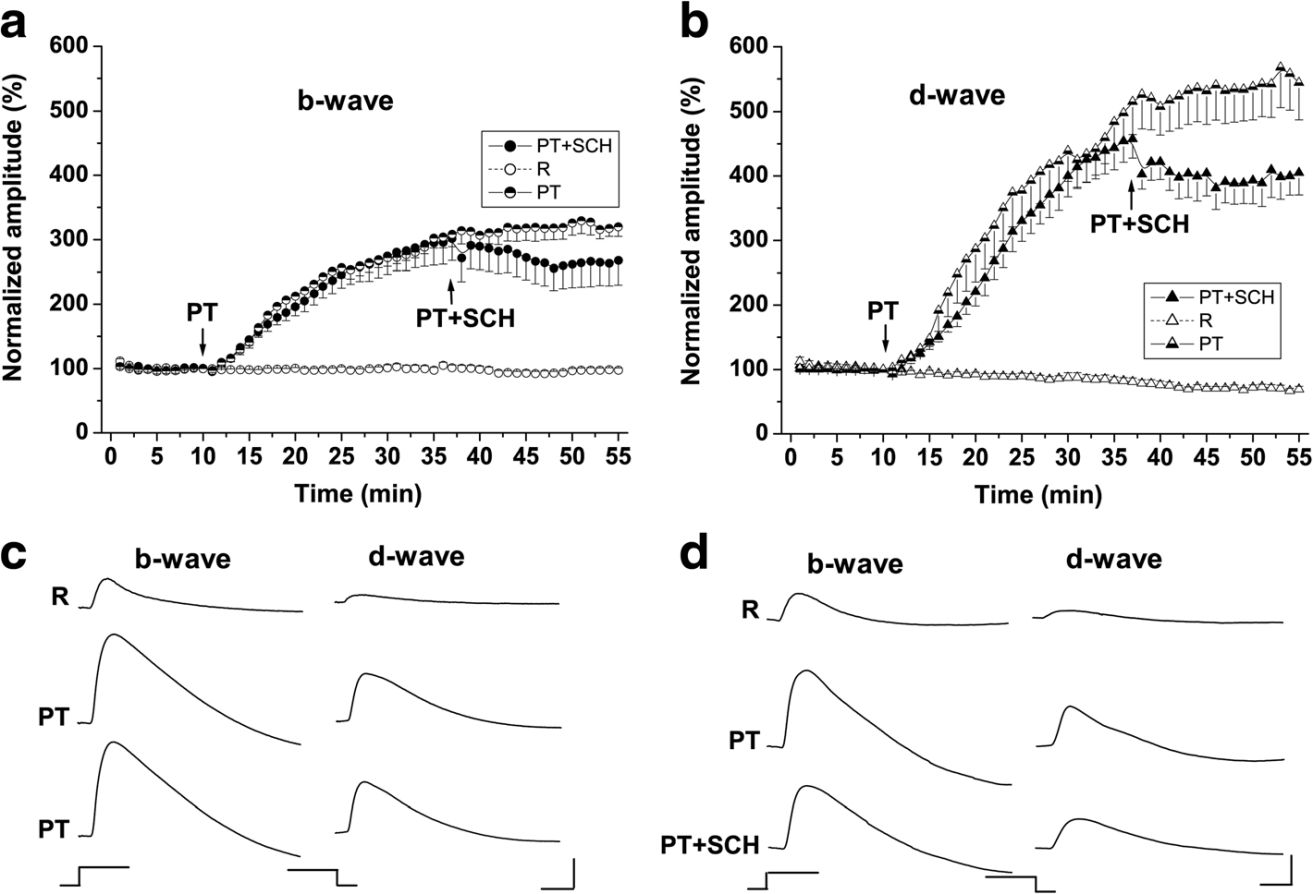
Effects of picrotoxin and picrotoxin plus SCH 23390 on the ERG waves **a**, **b** Time course of the effects of picrotoxin (PT) and picrotoxin plus SCH 23390 (PT + SCH) on the amplitudes of the ERG b- and d-waves. The results of different groups are indicated with open symbols in control experiments; hemi filled symbols in PT experiments and filled symbols in PT + SCH experiments. The amplitudes of the ERG waves were normalized to the values obtained just prior to picrotoxin application (10th minute from the beginning of the experiment). The times when the perfusion was switched to PT and PT + SCH are indicated by arrows. Mean values ± SEM are shown. **c** Original ERG records (b- and d-waves) obtained in the PT group during the perfusion with Ringer solution in the control period (10th minute from the beginning of the experiment - upper row) and during the perfusion with 50 μM picrotoxin: 37th minute (middle row) and 48th minute (bottom row) from the beginning of the experiment. The stimulus onset and offset are indicated below the traces. Calibration: time – 0.2 s; amplitude – 50 μV. **d** Original ERG records (b- and d-wave), obtained in the PT + SCH group during the perfusion with Ringer solution in the control period (10th minute from the beginning of the experiment - upper row), 50 μM picrotoxin (37th minute from the beginning of the experiment - middle row) and 50 μM picrotoxin + 75 μM SCH 23390 (48th minute from the beginning of the experiment - bottom row). The stimulus onset and offset are indicated below the traces. Calibration: time – 0.2 s; amplitude – 50 μV

The amplitude of the b-wave remained unchanged during the entire course of the control experiments. This is demonstrated in Figs. 1a and 2a, where the b-wave amplitude was normalized to the value (266 ± 56.12 μV) obtained in the 10th minute from the beginning of the experiments. The normalized d-wave amplitude (100% = 91 ± 27.7 μV) showed a tendency for small diminution, which is typical for the OFF responses in these stimulation conditions (Fig. 1b; Fig. 2b). The latency of the ERG waves was not significantly altered during the perfusion with Ringer solution (Table 1).

**Table 1:** Effects of SCH 23390, picrotoxin and combined picrotoxin + SCH 23390 application on the latency of the ERG b- and d-waves, obtained at different time periods (minutes from the beginning of the experiment)

Group	Latency (ms)	10 min	21 min	37 min	48 min
R	b-wave	115 ± 4.92	112 ± 6.58	111 ± 3.09	110 ± 5.20
	d-wave	104 ± 5.05	100 ± 7.14	105 ± 8.54	97 ± 8.45
SCH	b-wave	113 ± 3.47	117 ± 5.28		
	d-wave	95 ± 6.66	106 ± 8.77		
PT	b-wave	124 ± 7.36		135 ± 6.74	133 ± 7.21
				*p* < 0.008	
	d-wave	104 ± 6.91		127 ± 7.60	127 ± 7.29
				*p* < 0.00002	
PT+SCH	b-wave	127 ± 5.21		133 ± 5.98	133 ± 7.05
				*p* < 0.042	
	d-wave	112 ± 6.81		131 ± 7.60	127 ± 9.5
				*p* < 0.018	

The statistical significance of the differences between the values obtained before (10 min) and after drug application (37 min) is evaluated using paired *t*-test. Means ± SEM are shown

### SCH 23390 group

The amplitude of the ERG waves was relatively stable in the initial control period when the eyecups were perfused with Ringer solution only. Switching the perfusion to 75 μM SCH 23390 caused an increase of the amplitude of the b-wave (to ~ 127%) and d-wave (to ~ 137%) with respect to their initial values (100% = 195 ± 36.74 μV for the b-wave; 100% = 61 ± 13.18 μV for the d-wave) (Figs. 1 a, b). The effect developed rapidly and reached a plateau sooner for the b-wave (7th min from the beginning of SCH 23390 perfusion) than for the d-wave (11th min) (Figs. 1a, b). The effect of the blocker during the plateau period was very stable in time. There were no significant differences between the mean values of the normalized ERG wave amplitude at different time points during that period as revealed by Bonferroni post-hoc test and One-way repeated measures ANOVA with Greenhouse-Geisser correction (F _(1.65, 11.58)_ = 0.82, *p* > 0.05 for the b-wave; F_(2.1, 14.73)_ = 1.20, *p* > 0.05 for the d-wave). During the plateau period, the amplitudes of the b- and d-waves were significantly higher than the corresponding values obtained in the control group (Two-way ANOVA, F _(1,159)_ = 385.1, *p* < 0.001 for the b-wave; F _(1,159)_ = 433.4, *p* < 0.001 for the d-wave). Perfusion with SCH 23390 did not significantly alter the latency of the ERG waves (Table 1). These results are consistent with our previous findings in the dark adapted frog retina [15, 16] and indicate that endogenous dopamine acting through D_1_ receptors has a suppressive action on the amplitude of the ERG ON and OFF responses. The amplitude of the ERG waves recovered considerably when the perfusion was switched again to Ringer solution (Fig. 1 c).

### Picrotoxin group

Perfusion with 50 μM picrotoxin caused a considerable increase of the amplitude of the b-wave (~300%) and d-wave (~530%) in comparison to their initial values (b-wave: 100% = 263 ± 38.25 μV; d-wave: 100% = 108 ± 18.40 μV) (Figs. 2 a, b). The effect developed much slower than that of SCH 23390 and reached a plateau after 25 min from the beginning of picrotoxin perfusion. After that time, the effect was very stable till the end of the experiment (Figs. 2 a, b, c). No significant difference between the mean values of normalized ERG wave amplitude at different time points during the plateau period was observed as revealed by Bonferroni post-hoc test and One-way repeated measures ANOVA with Greenhouse-Geisser correction (F_(2.78, 22.28)_ = 2.63, *p* > 0.05 for the b-wave; F_(1.82, 14.58)_ = 1.02, *p* > 0.05 for the d-wave). During the plateau period, the amplitudes of both ERG waves were significantly greater compared to the corresponding values obtained in the control experiments (Two-way ANOVA, F _(1,157)_ = 1011.6, *p* < 0.0001 for the b-wave; F _(1,157)_ = 630.1, *p* < 0.001 for the d-wave). Picrotoxin not only altered the amplitude of the ERG responses, but also significantly delayed their latencies (Table 1). These results are in line with our previous data showing that the blockade of ionotropic GABA receptors in the frog retina always has an enhancing effect on the amplitude of the b- and d-waves combined with lengthening of their latencies irrespective of adaptation state and stimulus intensity used [13; for review: 3]. This means that activation of ionotropic GABA receptors by endogenous GABA leads to diminution of the amplitude, but speeds up the time courses of the ERG ON and OFF responses.

### Picrotoxin + SCH 23390 group

After the initial control period, the eyecups in this group were treated with picrotoxin alone for 27 min and with a combination of picrotoxin and SCH 23390 afterwards. As it could be expected from the results of the previous group, perfusion with picrotoxin alone caused a great enhancement to the amplitude of the b- wave (to ~ 300%; 100% = 194 ± 27.77 μV) and d-wave (to ~ 457%; 100% = 62 ± 9.2 μV) and lengthening of its response latency (Figs. 2 a, b, c) (Table 1). We compared the increase of the amplitude of the ERG waves in this group with that obtained in the picrotoxin group. No significant differences were obtained between the two groups (Two-way ANOVA, F _(1,130)_ = 0.66.6, *p* > 0.05 for the b-wave; F _(1,131)_ = 2.13, *p* >0.05 for the d-wave for the time between 30th and 37th minute from the beginning of the experiment). This indicates that the picrotoxin effect was expressed to a similar degree in both groups and thus, we could expect that it would have a similar time course. When the perfusion was switched to the solution containing picrotoxin and SCH 23390 (37th minute from the beginning of the experiment), a diminution of the amplitudes of the b- and d-waves was evident (Figs. 2 a, b). The effect developed rapidly and reached a plateau 8-10 min after the beginning of the picrotoxin + SCH 23390 perfusion. The plateau period was confirmed by Bonferroni post-hoc test and One-way repeated measures ANOVA with Greenhouse-Geisser correction (F_(2.76, 19.31)_ = 0.89, *p* > 0.05 for the b-wave; F_(2.30, 16.13)_ = 0.67, *p* > 0.05 for the d-wave), which revealed no significant differences between the mean values of normalized ERG wave amplitude at different time points during that period. The time course of the described effect resembled the time course of the effect of SCH 23390 alone, but was in the opposite direction. We compared the normalized amplitude values of the ERG waves during combined picrotoxin plus SCH 23390 treatment to its value obtained in picrotoxin group during the same time period (43 – 52 min). Two-way ANOVA revealed a significant difference between the two groups (F _(1,169)_ = 30.96, *p* < 0.0001 for the b-wave; F _(1,169)_ = 35.21, *p* < 0.0001 for the d-wave). This means that SCH 23390 applied on the background of the GABAergic blockade, significantly decreased the b- and d-wave amplitudes. The perfusion with picrotoxin + SCH 23390 did not significantly alter the latencies of the ERG responses as compared to that obtained during the preceding perfusion with picrotoxin alone (Table 1). This result could be expected because SCH 23390 alone also had no significant effect on this response parameter.

**Fig. 3:**
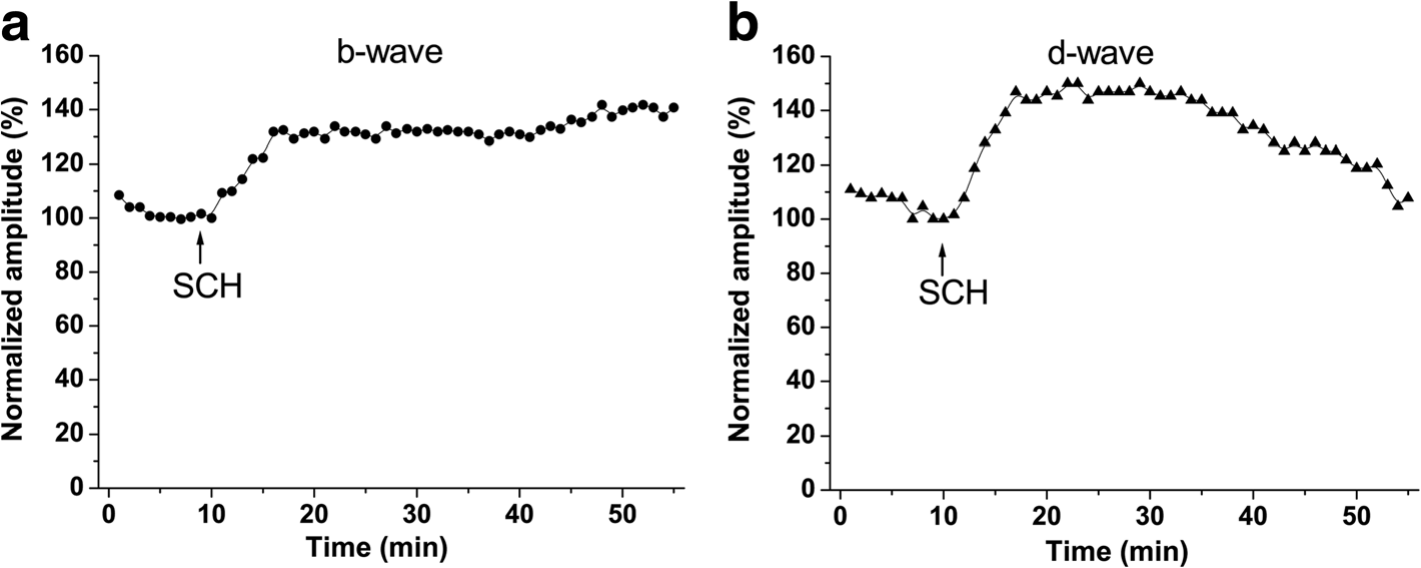
Effects of SCH 23390 on the ERG waves during long time application **a**, **b** Effects of SCH 23390 on the amplitudes of the ERG b- and d-waves during the long time period in one experiment. The amplitudes of the ERG waves were normalized to the values obtained just prior to SCH 23390 application. The times when the perfusion was switched to 75 μM SCH 23390 (SCH) are indicated by arrows

It was very interesting to investigate the interactions between the GABAergic system mediated by ionotropic GABA receptors and the dopaminergic system mediated by D_1_ receptors when their respective blockers were applied in the reverse order. However, the results from such experiments where SCH 23390 was applied first and then picrotoxin was co-applied could not be informative because the effects of SCH 23390 were not very stable at that time. The effects of SCH 23390 upon the d-wave amplitude declined after ~ 25 min from the beginning of the blocker perfusion (Fig. 3). If we switch the perfusion from SCH 23390 to SCH 23390 + picrotoxin after 11-12 min from the beginning of SCH 23390 perfusion (when the maximum of SCH effect is reached), we will only have 13-14 min with stable SCH 23390 effect upon the d-wave amplitude. During this short time period, picrotoxin effects could not reach their maximal expression. Thus, the effect of the GABAergic blockade could not be investigated on the background of stable D_1_ receptor blockade. As a consequence, no conclusion could be drawn for the interaction between the two systems (especially for the OFF response).

## Discussion

Our results clearly show that the isolated blockade of dopamine D_1_ receptors and ionotropic GABA receptors have enhancing effects on the amplitudes of both the ON and OFF responses in a frog ERG. This means that endogenous dopamine acting through D_1_-class receptors as well as endogenous GABA acting through ionotropic GABA receptors decreases the amplitudes of the ERG b- and d-waves. Our results are in odds with the results showing that the b-wave amplitude is suppressed in retinas with compromised D_1_ receptor function (*fish*: [17]; *rabbit*: [18, 19]; *mouse*: [10, 20–22]). The cited results indicate that the activation of D_1_ receptors by endogenous dopamine in fish, mouse and rabbit retinas enhances the b-wave amplitude. As the activities of ON bipolar cells are the main neuronal generators of the b-wave, it means that the light responses of ON bipolar cells are probably enhanced under the influence of dopamine D_1_ receptor activation. Unfortunately, the effects of selective D_1_ receptor agonists and antagonists on the light responses of single ON and OFF bipolar cells were not thoroughly investigated in different species. It has been shown, however, that dopamine, acting through D_1_-class dopamine receptors, enhances the response to glutamate in amphibian OFF bipolar cells [23]. As glutamate is the neurotransmitter of photoreceptors, it is reasonable to expect that the enhanced glutamate-gated current in OFF bipolar cells would enhance their light responses. A similar suggestion could be made from the results reported in the zebrafish retina, where dopamine decreases the voltage-activated potassium currents via D_1_ receptor-coupled G-protein pathways in ON bipolar cells [24]. All these indirect data led to the suggestion that activation of D_1_ receptors would enhance the light responses of both the ON and OFF bipolar cells.

If activation of D_1_ receptors has a similar action in the frog retina why then does SCH 23390 enhance rather than diminish the b- and d-wave amplitudes? One possible explanation is that dopamine, acting on D_1_ receptors, releases GABA from the GABAergic neurons. GABA would decrease the b- and d-wave amplitudes through its action on ionotropic GABA receptors. If ionotropic GABA receptors are blocked, however, this action of GABA would be prevented and dopamine D_1_ receptor activation would have an enhancing action on the ERG ON and OFF responses. We indeed demonstrated that the application of SCH 23390 during the ionotropic GABA receptor blockade caused a diminution instead of enhancement of the amplitude of both the b- and d-waves. Thus, it seems possible that the enhancing effect of D_1_ receptor activation on the ERG waves in the frog retina is obscured by the suppressive action of GABA released under the influence of dopamine. Dopamine D_1_ receptor inducing GABA release has been proposed to exist in the mouse retina [10] and is documented in the presence of free Ca^2+^ in the fish retina [25]. Because an opposite effect of dopamine on the GABA release was seen in Ca^2+^-free medium, it has been proposed that dopamine stimulates Ca^2+^-dependent GABA release and inhibits Ca^2+^-independent GABA release [25]. The latter suggestion is supported by other authors who have shown that dopamine acting through D_1_-class receptors fully inhibits the Ca^2+^-independent release of GABA in the fish retina [26–28]. Other authors insist, however, that dopamine only partly inhibits the Ca^2+^-independent release of GABA (*rat*:[29]) or that dopamine and D_1_ receptor agonist SKF 38393 inhibit the Ca^2+^-independent release of GABA only when NMDA receptors are activated (in Mg^2+^-free medium) (*chick*: [30]). An intriguing fact is that the latter effect of dopamine is not prevented by the antagonists of both dopamine receptor classes [30]. It was demonstrated [31] that dopamine and SKF 38393 bind to the NMDA channel and block it by a voltage-dependent mechanism in embryonic chick neurons. Similar effect has been seen in neonatal striatal neurons, but not in adult animals, where dopamine D_1_ receptor activation produces a potentiation of the NMDA response [32]. It remains to be determined if similar developmental switch in D_1_ modulation of NMDA receptors occurs in the retina. More studies are needed to understand the effect of dopamine D_1_ receptor activation on GABA release and GABAergic neurotransmission in vertebrate retina.

## Conclusion

Our results clearly show that an isolated D_1_ dopamine receptor blockade by SCH 23390 as well as an isolated ionotropic GABA receptor blockade by picrotoxin has an enhancing effect on the b- and d-wave amplitudes in a frog ERG. The enhancing effect of D_1_ receptor blockade is not seen during simultaneous application of picrotoxin, indicating that there is a significant interaction between dopaminergic and GABAergic systems in the frog retina.

## Additional file

Additional file 1: Figure S1. Effects of different concentrations of SCH 23390 on the amplitudes of the ERG b- and d-waves. The amplitudes of the ERG waves were normalized to the values obtained just prior to SCH 23390 application. Means ± SEM are shown. (TIFF 879 kb)
