# Supplementary figures and images for: Effects of dopamine D1 receptor blockade on the ERG b- and d-waves during blockade of ionotropic GABA receptors
Source: Eye Vis (Lond). 2016 Dec 7;3:32. doi: 10.1186/s40662-016-0064-4 (PMC5142325; doi:10.1186/s40662-016-0064-4)

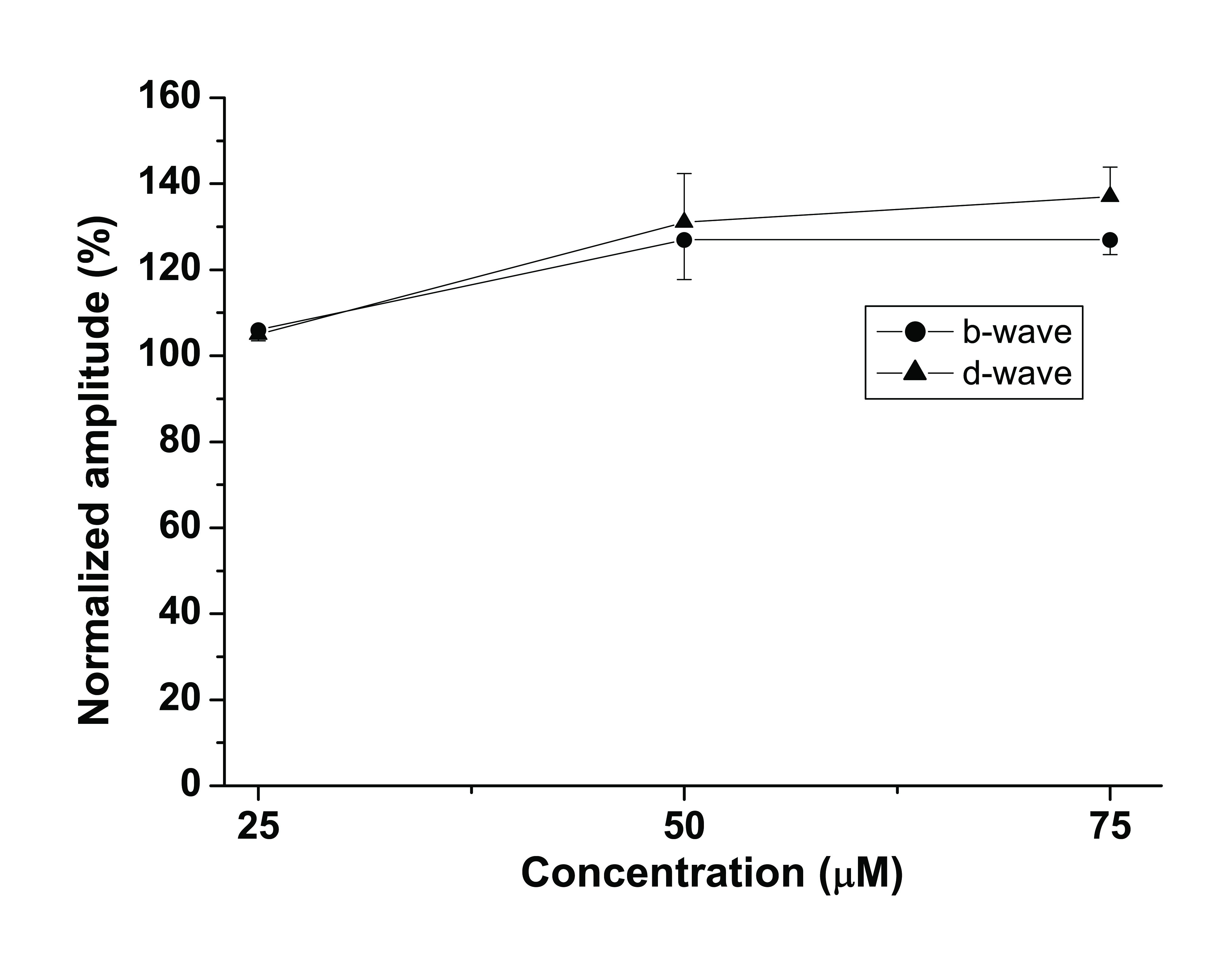

Supplement: Additional file 1: Figure S1. — Effects of different concentrations of SCH 23390 on the amplitudes of the ERG b- and d-waves. The amplitudes of the ERG waves were normalized to the values obtained just prior to SCH 23390 application. Means ± SEM are shown. (TIFF 879 kb) [file 40662_2016_64_MOESM1_ESM.tiff]
